# The efficacy of an online exercise intervention for improving depressive symptoms among patients with subthreshold depression in primary care: protocol for a randomized controlled trial

**DOI:** 10.1186/s12888-025-06663-0

**Published:** 2025-04-09

**Authors:** Ken O. T. Yu, Eric K. P. Lee, Benjamin H. K. Yip, Dicken C. C. Chan, Winnie W. S. Mak, Tatia M. C. Lee, Wai Kwong Tang, Maria K. W. Leung, Stanley S. C. Hui, Samuel Y. S. Wong

**Affiliations:** 1 Department of Psychiatry, The Chinese University of Hong Kong, Hong Kong, China; 2 Department of Psychology, The University of Hong Kong, Hong Kong, China; 3 Department of Sports Science and Physical Education, The Chinese University of Hong Kong, Hong Kong, China; 4 JC School of Public Health and Primary Care, The Chinese University of Hong Kong, Hong Kong, China; 5 Department of Family Medicine & Primary Health Care, New Territories East Cluster, Hospital Authority, Hong Kong, China; 6 Department of Psychology, The Chinese University of Hong Kong, Hong Kong, China

## Abstract

**Background:**

Subthreshold depression is common in primary care and is associated with significant healthcare burden. There is emerging evidence for the benefits of Exercise can reduce depressive symptoms among people with diagnosed depression, but there is limited evidence for subthreshold depression in primary care setting. This study aims to examine the efficacy of a 12-week online instructor-led exercise intervention in reducing depressive symptoms among people with subthreshold depression in primary care, when compared to usual care control.

**Methods:**

This 1:1 randomised controlled trial will enrol 260 participants with subthreshold depression randomizing into 2 groups (online exercise intervention versus usual care control). The intervention consists of twice-weekly 1-h exercise online classes over 12 weeks, which will be led by certified instructors. Data will be collected at baseline (T0), immediate post intervention (T1), 3-month post intervention (T2) and 9-month post intervention (T3). The primary outcome is depressive symptoms measured by the Chinese version of Beck Depression Inventory-II (BDI-II) at T1. Secondary outcomes include anxiety symptoms, quality of life, physical activity levels, feasibility and acceptability, medication use and health service utilization, and cost assessment. Intention-to-treat analysis will be performed.

**Discussion:**

The proposed study will assess the efficacy of online exercise intervention in improving subthreshold depressive symptoms in primary care. The findings will inform clinicians and policy makers concerning prevention of depression in primary care, and may lead to changes in the respective current guidelines and public policies.

**Trial Registration:**

This trial has been registered in the Chinese Clinical Trial Registry, with the registration number: ChiCTR2400087923. The date of registration is 2024–08-07.

**Supplementary Information:**

The online version contains supplementary material available at 10.1186/s12888-025-06663-0.

## Introduction

### Background

Subthreshold symptoms that do not fulfil the diagnostic criteria for major depression are common in primary care [1] and are associated with significant healthcare burden [2]. People with subthreshold depression are at high risk for developing major depression with an incidence of 18%, three times as much as that in the general population [3]. In addition, up to 60% may have a chronic course with 25% developing into a more severe form of depression [4]. Therefore, evidence-based interventions that can be readily implemented in primary care are needed.

At present, cognitive behavioural therapy (CBT) or CBT-based problem-solving therapy is the most evidence-based interventions for people with subthreshold depressive symptoms [5]. However, there is often a long waiting time for CBT-based interventions in the public sector while the cost for psychotherapy can be prohibitive in the private sector. Moreover, people at increased risk for depression including older adults and people of disadvantaged background may either lack the cognitive skills or the time needed for CBT [6]. On the other hand, primary care providers may find CBT training and interventions too time consuming. As the demand for evidence-based psychological interventions is far greater than services available in Hong Kong, a simple and implementable preventive intervention for managing depressive symptoms is urgently needed.

It is well established that physical exercise is beneficial to health. Evidence shows that exercise can improve mood and reduce depression and anxiety in addition to physical health benefits [7]. A recent large overview of 97 systematic reviews on the effectiveness of exercise interventions for improving depression, anxiety and psychological distress showed that exercise can improve depressive symptoms across various adult populations including older adults and people with depressive symptoms, demonstrating a moderate to large effect size similar to that of psychotherapy and pharmacotherapy [8].

Despite the well-proven health benefits of exercise, physical inactivity is common. A recent Hong Kong survey shows that about 25% of adults aged 18 or above had insufficient physical activity according to the World Health Organization (WHO) recommendation [9]. Moreover, there is a significant increase in the prevalence of physical inactivity when compared to findings from a previous survey [10]. Among people with chronic conditions including depression, only about 25% participate in regular physical activities.

The WHO has recommended reducing physical inactivity as one of the “best buy” policies in tackling non-communicable diseases [11]. However, patients face significant barriers to engage in physical activity including lack of knowledge, skills and resources (e.g. venue and materials for exercise) [12, 13], lack of social support [13], receiving non-specific instructions from health care professionals (for patients) [12] and not considering physical exercise as important [12]. From the theory of behavioural economics [14], people's preferences are highly malleable and seemingly trivial contextual factors such as availability of venues, or the presence of simple feedback can have substantial influence on people’ health behaviours. This is supported by research showing that effects from facilitated physical activity based on health coaching alone using physical activity facilitators (no physical trainers to provide instructions or skills transfer) may not be large enough to produce clinically significant effects on depressive symptoms [15]. Simple behavioural techniques [16] such as instruction on how to perform a behaviour, followed by feedback on behaviours and the provision of a clear pathway in identifying context, frequency, duration and intensity of the required behaviours can all substantially result in positive changes of desirable behaviours and improvement of health outcomes.

Previously, a clinical trial was conducted to examine the effectiveness of a complex exercise programme, named the “Exercise Is Medicine” (EIM) programme, in normalizing the nocturnal blood pressure of hypertensive patients in Hong Kong [17]. Due to the social restriction policy implemented in Hong Kong during the peak of COVID-19 pandemic, part of the EIM programme was delivered through the Zoom online platform. Although depressive symptoms were not the outcome of the study, such symptoms (in the form of Patient Health Questionnaire depression scale [PHQ-9] [18]) were measured for all participants. Fifty-four people with mild depressive symptoms at baseline received online exercise intervention. Class attendance rate was 90.4% while dropout rate at 12 month follow up was 13%. The preliminary results (yet to be published, by courtesy of the authors) showed a significant decrease of mean PHQ-9 score from 6.7 at baseline to 3.8 at 12 months after recruitment (95% confidence interval for the mean difference was −4.1, −1.7). Although these preliminary findings are encouraging, the exploratory nature and small sample size resulted in limited internal validity. A properly designed randomised controlled trial is therefore needed to examine the programme’s efficacy in reducing depressive symptoms among patients with subthreshold depression when compared to a control group.

Furthermore, while there are a number of trials evaluating the cost-effectiveness of various internet-based interventions for the management of depression [19], none of the trials included online exercise programme in their intervention arms. Thus, a new study focusing on an internet-based, structured exercise intervention is needed to provide data for preliminary cost assessment and insights into the cost-effectiveness of such intervention in primary care setting.

### Objectives and Hypotheses

The primary objective of the study is:

To examine the efficacy of a 12-week online instructor-led exercise intervention (EIM) when compared to usual care control in reducing depressive symptoms among people with subthreshold depression in primary care.

The secondary objectives of the study are:

- To examine the efficacy of a 12-week online instructor-led exercise intervention (EIM) when compared to usual care control in reducing anxiety symptoms and improving quality of life among people with subthreshold depression in primary care; and
- To examine the cost-effectiveness of the intervention.

The hypotheses of the study are:

- The online instructor-led exercise intervention will be more effective when compared to usual care in reducing depressive symptoms among patients with subthreshold depression (primary hypothesis);
- The online instructor-led exercise intervention will be more effective in reducing anxiety symptoms and improving quality of life when compared to usual care (secondary hypothesis); and
- The online instructor-led exercise intervention will be a cost-effective intervention in primary care (secondary hypothesis).

## Methods

### Study design

**Fig. 1:**
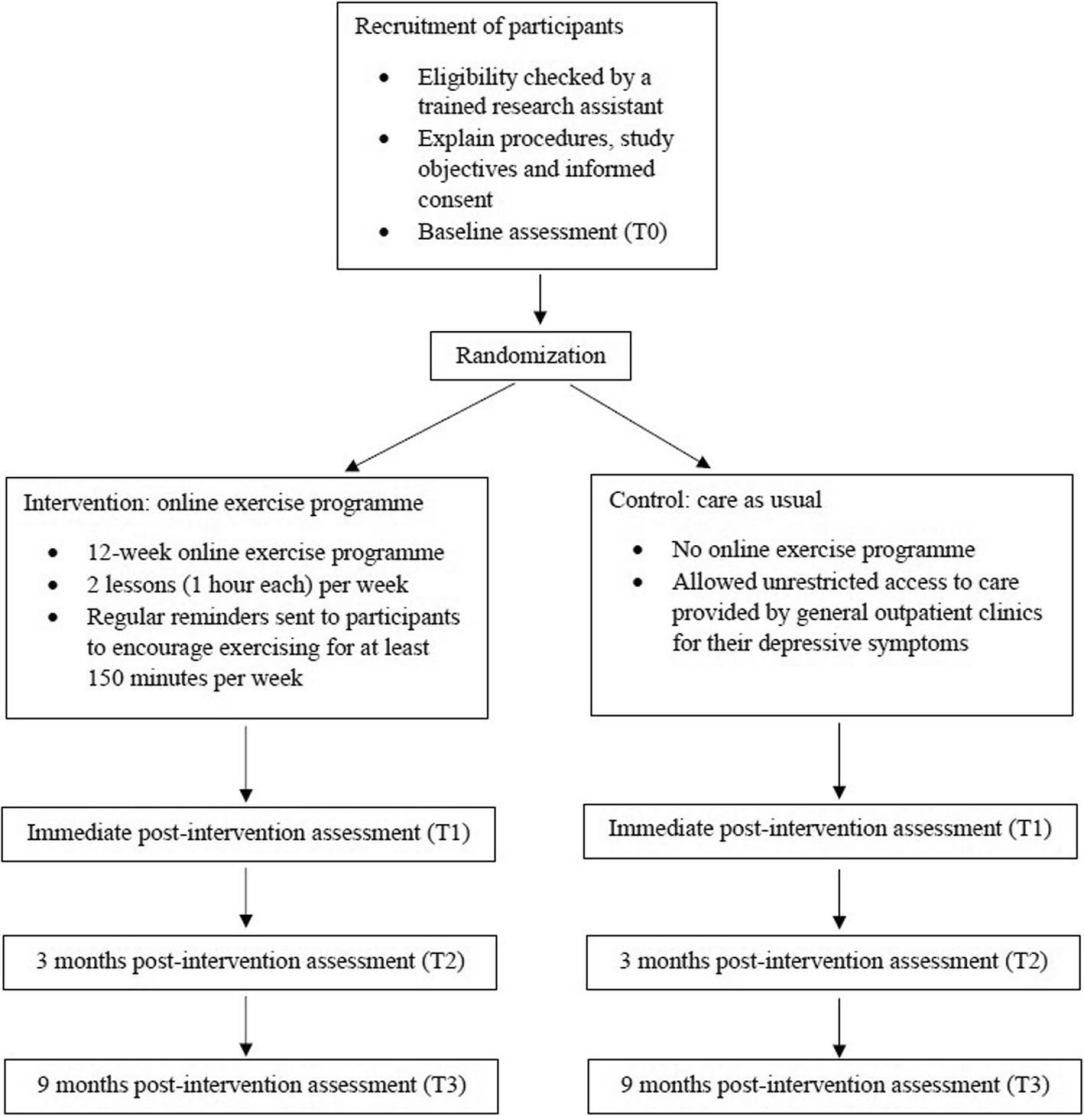
Schematic overview of the study. Caption: Participants will be randomized into online exercise programme group and control group. Assessments will be conducted at baseline (T0), immediate post intervention (T1), 3-month post intervention (T2) and 9-month post intervention (T3)

This will be a pragmatic randomized controlled trial with two arms: online instructor-led exercise intervention versus usual care control in primary care. Assessments will be conducted at baseline (T0), immediate post intervention (T1), 3-month post intervention (T2) and 9-month post intervention (T3). An overview of the study design is shown in Fig. 1.

### Participants

The following inclusion and exclusion criteria will be used to determine the eligibility of the participants.

The inclusion criteria are: (1) aged 18 years or above; and (2) having subthreshold depression as defined by having a score 5–9 on the Patient Health Questionnaire depression scale (PHQ-9) [18], i.e., having a total score of 5 to 9 inclusive.

The exclusion criteria are: (1) dysthymia with subthreshold depressive symptoms that last for 2 years or more; (2) major depression within the past 6 months; (3) lifetime history of other psychiatric disorders (including but not limited to generalized anxiety disorder, schizophrenia, bipolar affective disorder and substance use disorder); (4) current use of antidepressant; (5) currently enrolled in any form of psychological interventions for any depressive symptoms; (6) medical illness with a prognosis of less than 6 months to live; and (7) recently diagnosed or uncontrolled cardiovascular, metabolic or neurological conditions.

### Study setting and recruitment

Participants will be recruited from public primary care clinics, i.e., General Outpatient Clinics (GOPCs) and Family Medicine Clinics (FMCs) of the New Territories East Cluster (NTEC) of Hong Kong. Currently, GOPCs and FMCs in NTEC serve a population of more than 1 million residents, resulting in a high representativeness. Apart from GOPCs and FMCs, recruitment will also be conducted in collaboration with non-governmental organizations, which will refer individuals with primary care needs in the community.

### Intervention: Online exercise programme

The design of the exercise class (duration and frequency) is modified from the class for diabetes and hypertension based on findings from recent systematic reviews on the effectiveness of physical activity intervention for improving depression and anxiety [8]. The 12-week EIM program aims to transform participants' exercise habits by fostering and maintaining behavioural changes through motivation, as well as providing relevant knowledge and experience. The online classes will be conducted by certified physical trainers who hold a post-secondary degree (or equivalent), possess a valid basic life support certificate, and have been certified by the Physical Fitness Association of Hong Kong after passing examinations. They have also completed specialized training to deliver the EIM program.

The 12-week EIM online class on the Zoom platform (details in Appendix 1) will enrol 11 to 18 patients per group and meet twice a week for 1 h each time. Each class contains a set of exercises including stretching, aerobic exercise and resistance training (combined exercise) and will be carried out according to specific protocol [17] (Appendix 1). Participants will receive reminders about each session via WhatsApp or telephone calls. After each class, home exercises were assigned to achieve a minimum of 150 min of exercise per week. Additionally, regular SMS or WhatsApp messages will be sent to remind and encourage participants to incorporate exercise into their daily routines. Furthermore, participants will also be provided with smartwatches to track their physical activities. The trainers can monitor participants' activity levels via trackers before each lesson, enabling them to motivate individuals and address barriers as needed, thereby offering regular reinforcement, social support, and problem-solving assistance. Zoom lessons will be recorded, and approximately 15% of these recordings will be reviewed by the investigator to ensure fidelity to the intervention.

### Care as usual control

Participants in the both groups have unrestricted access to GOPCs for any health problem, including depressive symptoms. In Hong Kong, the general outpatient clinics provide primary care services at a low subsidized fee.

Participants assigned to the control group will receive medical care from GOPCs. In Hong Kong, individuals with subthreshold depression are managed in primary care setting instead of being referred to mental health specialists.

### Randomization, concealment and blindness

Stratified randomization will be used to enhance balance of gender, anxiety symptoms (“minimal to mild” versus “moderate to severe” as classified by the Beck Anxiety Inventory [21]) and level of physical activity (“low”, “medium” and “high” as classified by the International Physical Activity Questionnaire – Short Form [23]) at baseline between two arms. The randomisation procedure will be conducted by an independent statistician. Participants will be individually notified of the randomization results via phone or WhatsApp messages by a research assistant. Outcome assessors will be blinded from the group assignment. Due to the characteristics of the intervention, researchers and participants cannot be blinded after group allocation.

### Data collection

Demographic information, including gender, age, education level, marital status and household income, will be collected at T0. All outcomes will be measured at T0, T1, T2 and T3 (Fig. 1). The primary and secondary outcome measures are described in details below:

#### Primary outcome

T1 will be chosen as the primary outcome endpoint as it represents the time at which we expect to observe the largest effect of intervention. Self-reported depressive symptoms will be measured by the validated the Chinese version Beck Depression Inventory-II (BDI-II) [20] which is more sensitive than PHQ-9 in measuring changes in depressive symptoms in clinical studies. The BDI-II is a 21-item inventory of depressive symptoms. The internal consistency and factor structure of the BDI-II has received extensive support among outpatient samples of adults which indicates that it is a very reliable and well validated scale to measure depressive symptom severity [20]. It is the premier instrument for the assessment of depressive symptom severity and the tracking of short-term changes in severity in outpatient settings.

#### Secondary outcomes

##### Anxiety symptoms

Anxiety symptoms will be measured by the Beck Anxiety Inventory [21], which is a self-report measure consisting of 21 items rated on a Likert scale from 0 to 3, representing ‘not at all’ to ‘severely’. It has satisfactory internal consistency in the Chinese population [21].

##### Quality of life

Quality of life will be measured by the Chinese version Medical Outcomes Study Short-Form Health Survey (SF-12) [22]. The SF-12 is a 12-item survey that measures health-related quality of life, including both physical and mental functioning and well-being. It is validated in local Chinese population [22].

##### Physical activity levels

Physical activity levels will be measured by the International Physical Activity Questionnaire – Short Form (IPAQ-SF) [23], which is a reliable and valid in the local Hong Kong population. The original IPAQ assesses the amount of exercise, physical activities and sitting time in the last 7 days. In addition, physical activity data obtained by the wrist trackers will be used to validate the responses to the IPAQ-SF. If there is any discrepancy, the research assistant will contact the participants for verification. We will further ask if their exercise habit in the last 7 days is different than their usual exercise amount.

##### Feasibility and acceptability

Recruitment rate will be determined by the ratio between the total number of participants randomised and the total number of persons meeting inclusion criteria. Feasibility will be examined by tracing class attendance and retention, supplemented with data of the participants’ physical activity levels recorded from the wearable trackers. Acceptance will be assessed by collecting evaluation form immediately after the intervention (Appendix 2).

##### Medication use and health service utilization

All medications associated with an intention to improve mood or mood-related symptoms will be recorded at follow-up. These include anticonvulsants, antidepressants and anxiolytics including benzodiazepines. Utilization of health services including visits to primary care doctors (both private and public) or accident and emergency units, visits to any specialist outpatient clinics, use of other psychiatric services such as psychologists, social workers or occupational therapists, and hospitalizations will be recorded at all visits to see whether there are changes between groups on health services utilization. The electronic patient records of the Hospital Authority for the utilization of public healthcare services will be checked, supplemented by the participants’ self-report.

##### Cost assessment

Costs using both provider and societal perspectives will be collected. The cost of healthcare service, the productivity loss of the participants and cost of caregiver will be self-reported by the participants. The cost of productivity loss of the participants will be estimated by the number of sick leave and the cost of each working days. The frequency of utilizing healthcare service and the cost of each visit including medication and transportation fee will be recorded. The cost of caregiver will be the money and time spent on taking care of the participants by caregivers. The cost of the online exercise intervention includes hiring cost of physical trainers, other administrative costs associated with the operation of the online exercise intervention programme, and costs of purchasing and maintaining the wearable trackers. The data from cost assessment will be used with the BDI-II and SF-12 scores for evaluating the cost-effectiveness of the online exercise programme.

### Sample size

From a previous trial on assessing the efficacy of group-based behavioural activation with mindfulness for treating subthreshold depression in primary care in Hong Kong, mean (standard deviation) of BDI-II score at baseline for intervention and usual group were 19.18 (8.35) and 17.68 (9.52) respectively among participants with subthreshold depression. Pooled mean and standard deviation were 18.43 and 8.95 from two groups. For moderate baseline severity, the minimum clinically important difference (MCID) on the BDI-II was estimated at 3.5 points [24]. Assuming at least the MCID could be seen in the online exercise programme at 4-month post randomisation compared to usual care, sample size of 104 each group achieve 80% power to detect the difference of 3.5 points in BDI-II with a common standard deviation of 8.95 at a significance level of 0.05 using two-sided t-test. The corresponding effect size of 0.39 is similar to the finding from a systematic review that physical activity is beneficial for improving depressive symptoms with effect size of 0.43 [8]. From our experience in running online exercise intervention for people with subthreshold depression, a dropout rate of 13% at 12 month follow up was observed. To be conservative, we assume drop out to be 20%, and set our enrolment target at participants in total of 260 with 130 participants in each group.

### Statistical analysis

Baseline characteristics of the two groups will be compared using chi-square test for categorical variables and independent samples t-test for continuous variables. Analysis of covariance (ANCOVA) for the BDI-II score at T1 will be conducted as the primary analysis, with the BDI-II score at T0 and other characteristics with significant between-group difference at T0 as covariates in the model for sensitivity analyses. Effect size estimates will be obtained for the primary outcome, with the between group effect sizes (Cohen’s d) being calculated using the mean differences from baseline and the SDs of the two groups at each time point. For the secondary analysis, linear mixed models (LMM) with random intercept will be applied to investigate the significant changes over time, for both primary and secondary outcome measures. The LMM approach is in line with the intention-to-treat principle, to include all subjects for the analysis and use all available data to assess the treatment effect over time. Maximum likelihood estimation (MLE) will be employed to handle missing data in the analyses with LMMs. A sensitivity analysis will be conducted using imputed data generated by multiple imputation. Furthermore, compliance with prescribed exercises will be added into the models to explore if this is predictor of changes in health outcomes.

In the cost-utility analysis, utility will be measured with quality adjusted life years (QALYs) based on SF-12 using the Hong Kong weights. The area under curve approach will be used to calculate the utilities at follow-ups after adjusting for baseline utility. In the cost-effectiveness analysis, remission of depressive symptoms (BDI-II score of < 14) will be used as outcome. Using both provider and societal perspectives, incremental cost-effectiveness ratio (ICER) will be calculated by dividing cost difference of two groups by between-group effect difference. We will use bootstrapping technique to assess the uncertainty of the ICER estimate. Due to the lack of a local willingness-to-pay threshold, we will generate cost-effectiveness acceptability curves to evaluate the probability of cost-effectiveness of the proposed intervention.

The significant level will be set as 0.05 for the statistical tests. To address the issue of multiple testing in our exploratory secondary analyses, we will employ the Bonferroni correction to control the overall type I error rate by adjusting the significance threshold for each individual test. This adjustment ensures a rigorous control of false positive results while allowing for the exploration of associations between the online exercise intervention and secondary outcomes.

### Ethical concerns and data monitoring

Participants will be informed of the details about this study, including the objectives, procedures, randomization arrangement, and schedule of intervention and data collection. Research staff will allow ample time for the potential participants to decide whether they opt to join the study or not. Written consent will be obtained from all participants. Participants can choose to withdraw from the study at any time. In case of any adverse event, the ethics committee will be notified immediately. Moreover, all participants will be covered by clinical trial insurance. Qualified physical trainers with adequate knowledge and experience to assess participants’ physical health and fitness levels will be hired to lead the intervention. They will help identify any potential risks to ensure the exercise activities are performed safely.

In addition, a data and safety monitoring committee will be established consisting of a biostatistician being the chair of the committee, one expert in mental health and one expert in public health to monitor the study outcomes on a regular basis to ensure there is no safety issues arise from the study. All committee members will be independent from the study. The committee can decide to stop the trial if there are strong evidence of safety issue caused by intervention. To ensure subject privacy and research data confidentiality, all hardcopies of the questionnaires and data collected will be locked in cabinets, and their electronic versions will be stored in password protected computers.

## Discussion

Numerous studies have confirmed the benefits of exercise in reducing depressive symptoms among people with diagnosed depression, but only limited studies [25] were conducted in primary care. Moreover, most have short follow-ups and few explored the effectiveness of exercise intervention among patients with subthreshold depression and examined the prevention of having clinically relevant depressive symptoms or major depression [8]. In addition, as the COVID pandemic has prompted the rapid development and adoption of Zoom conducted exercise programme and there are limited studies using online exercise intervention for depressive symptoms [26], this also provides a unique opportunity to examine the efficacy of Zoom exercise programme.

A study in Sweden [19] showed that physical exercise intervention was a cost-effective treatment for mild to moderate depression in primary care setting compared to treatment as usual, with an incremental cost per quality-adjusted life-year (QALY) gain of 14,571 euros. However, the same study also found that internet-based cognitive behavioural therapy (ICBT) resulted in a lower incremental cost per QALY gain of 8,817 euros. One of the possible reasons for the discrepancy is that the physical exercise intervention required face-to-face sessions, which resulted in transportation cost and cost of securing venues for the sessions. In contrast, such costs were not incurred in the ICBT arm. If the physical exercise intervention were converted to an online format, the cost-effectiveness might be improved.

Therefore, we propose to examine the efficacy and cost-effectiveness of a complex intervention (EIM) using Zoom supported by other components in reducing depressive symptoms among patients with subthreshold depression in primary care. Future effectiveness study can be conducted to determine whether the intervention produces the proven efficacy in real-world setting.

## Publication and data availability

The results of this study will be published in peer-reviewed journals and presented in public conferences, in accordance to the CONSORT-2010 standards. The datasets generated and/or analysed during the study will be available from the corresponding author upon reasonable request following the completion of all primary publications derived from these datasets.

## Supplementary Information

Supplementary Material 1.

Supplementary Material 2.

Supplementary Material 3.

## Data Availability

No datasets were generated or analysed during the current study.

## Abbreviations

ANCOVA: Analysis of covariance
BAI: Beck Anxiety Inventory
BDI-II: Beck Depression Inventory-II
CBT: Cognitive behavioural therapy
EIM: “Exercise Is Medicine” programme
FMC: Family Medicine Centre
GOPC: General Outpatient Clinic
ICER: Incremental cost-effectiveness ratio
IPAQ-SF: International Physical Activity Questionnaire – Short Form
LMM: Linear mixed model
MCID: Minimum clinically important difference
MLE: Maximum likelihood estimation
NTEC: New Territories East Cluster, Hospital Authority, Hong Kong
PHQ-9: Patient Health Questionnaire-9
QALY: Quality adjusted life year
SF-12: Medical Outcomes Study Short-Form Health Survey

## References

[1] Rucci P, Gherardi S, Tansella M, Piccinelli M, Berardi D, Bisoffi G, et al. Subthreshold psychiatric disorders in primary care: prevalence and associated characteristics. J Affect Disord. 2003;76(1–3):171–81. 12943947 10.1016/s0165-0327(02)00087-3

[2] Cuijpers P, Smit F, Oostenbrink J, de Graaf R, Ten Have M, Beekman A. Economic costs of minor depression: a population-based study. Acta Psychiatr Scand. 2007;115(3):229–36. 17302623 10.1111/j.1600-0447.2006.00851.x

[3] Zhang R, Peng X, Song X, et al. The prevalence and risk of developing major depression among individuals with subthreshold depression in the general population. Psychol Med. 2023;53(8):3611–20. 10.1017/S0033291722000241. 10.1017/S0033291722000241 PMC10277767 35156595

[4] Spek V, Cuijpers P, Nyklicek I, et al. One year follow-up results of a randomized controlled clinical trial on internet-based cognitive behavioural therapy for subthreshold depression in people over 50 years. Psychol Med. 2008;38:635–9. 18205965 10.1017/S0033291707002590

[5] National Institute for Health and Care Excellence. Depression in adults: treatment and management. London: NICE; 2022. Available from: https://www.nice.org.uk/guidance/ng222. 35977056

[6] Fiske A, Wetherell JL, Gatz M. Depression in older adults. Annu Rev Clin Psychol. 2009;5:363–89. 19327033 10.1146/annurev.clinpsy.032408.153621 PMC2852580

[7] Mikkelsen K, Stojanovska L, Polenakovic M, Bosevski M, Apostolopoulos V. Exercise and mental health. Maturitas. 2017;106:48–56. 29150166 10.1016/j.maturitas.2017.09.003

[8] Singh B, Olds T, Curtis R, et al. Effectiveness of physical activity interventions for improving depression, anxiety and distress: an overview of systematic reviews. Br J Sports Med. 2023;57(18):1203–9. 10.1136/bjsports-2022-106195. 10.1136/bjsports-2022-106195 PMC10579187 36796860

[9] Centre for Health Protection. Report of Population Health Survey 2020–22 (Part I). Centre for Health Protection, Department of Health, the Government of the Hong Kong SAR; 2022. Accessed February 24, 2023.https://www.chp.gov.hk/files/pdf/dh_phs2020-22_part_1_full_report_eng_20221222.pdf.

[10] Centre for Health Protection. Report of Health Behaviour Survey 2018/19. Centre for Health Protection, Department of Health, the Government of the Hong Kong SAR; 2020. Accessed February 24, 2023. https://www.chp.gov.hk/files/pdf/report_of_health_behaviour_survey_2018_en.pdf.

[11] World Health Organization. “Best Buys” and other Recommended Interventions for the Prevention and Control of Noncommunicable Diseases. World Health Organization; 2017. Accessed February 28, 2023. https://www3.paho.org/hq/dmdocuments/2017/ents-best-buys-english.pdf.

[12] Eakin E, Brown W, Schofield G, Mummery K, Reeves M. General practitioner advice on physical activity-who gets it? Am J Health Promot. 2007;21(4):225–8. 17375487 10.4278/0890-1171-21.4.225

[13] Sit CHP, Kerr JH, Wong ITF. Motives for and barriers to physical activity participation in middle-aged Chinese women. Psychol Sport Exerc. 2008;9(3):266–83.

[14] Thorgeirsson T, Kawachi I. Behavioral economics: merging psychology and economics for lifestyle interventions. Am J Prev Med. 2013;44(2):185–9. 23332337 10.1016/j.amepre.2012.10.008

[15] Chalder M, Wiles NJ, Campbell J, et al. Facilitated physical activity as a treatment for depressed adults: randomised controlled trial. BMJ. 2012;344: e2758. 22674921 10.1136/bmj.e2758 PMC3368484

[16] King AC, Blair SN, Bild DE, et al. Determinants of physical activity and interventions in adults. Med Sci Sports Exerc. 1992;24(6 Suppl):S221–36. 1625548

[17] Lee EK, Zhang DD, Yip BH, et al. Exercise Intervention to Normalize Blood Pressure and Nocturnal Dipping in HyperTensive Patients (END-HT): Protocol of a Randomized Controlled Trial. Am J Hypertens. 2021;34(7):753–9. 33471104 10.1093/ajh/hpab019

[18] Kroenke K, Spitzer RL, Williams JB. The PHQ-9: validity of a brief depression severity measure. J Gen Intern Med. 2001;16(9):606–13. 11556941 10.1046/j.1525-1497.2001.016009606.x PMC1495268

[19] Kraepelien M, Mattsson S, Hedman-Lagerlöf E, Petersson IF, Forsell Y, Lindefors N, et al. Cost-effectiveness of internet-based cognitive–behavioural therapy and physical exercise for depression. BJPsych Open. 2018;4(4):265–73. 10.1192/bjo.2018.38. 30057780 10.1192/bjo.2018.38 PMC6060490

[20] Shek DT. Reliability and factorial structure of the Chinese version of the Beck Depression Inventory. J Clin Psychol. 1990; 46(1): 35–43.33. Joiner TE Jr, Walker RL, Pettit JW, Perez M, Cukrowicz KC. Evidence-based assessment of depression in adults. *Psychol Assess*. 2005;17(3):267–277. 10.1002/1097-4679(199001)46:1<35::aid-jclp2270460106>3.0.co;2-w 2303562

[21] Cheng SK, Wong CW, Wong KC, et al. A study of psychometric properties, normative scores, and factor structure of the Beck Anxiety Inventory–the Chinese version. Chinese J Clin Psychol. 2002;10:4–6.

[22] Lam CLK, Tse EYY, Gandek B. Is the standard SF-12 health survey valid and equivalent for a Chinese population. Qual Life Res. 2005;14:539–47. 15892443 10.1007/s11136-004-0704-3

[23] Cerin E, Barnett A, Cheung MC, Sit CHP, Macfarlane DJ, Chan WM. Reliability and validity of the IPAQ-L in a sample of Hong Kong urban older adults: does neighborhood of residence matter? J Aging Phys Act. 2012;20(4):402–20. 22186607 10.1123/japa.20.4.402

[24] Kounali D, Button KS, Lewis G, et al. How much change is enough? Evidence from a longitudinal study on depression in UK primary care. Psychol Med. 2022;52(10):1875–82. 33138872 10.1017/S0033291720003700 PMC9340848

[25] Helgadóttir B, Forsell Y, Hallgren M, Möller J, Ekblom Ö. Long-term effects of exercise at different intensity levels on depression: A randomized controlled trial. Prev Med. 2017;105:37–46. 28823684 10.1016/j.ypmed.2017.08.008

[26] Li J, Theng YL, Foo S. Play mode effect of exergames on subthreshold depression older adults: a randomized pilot trial. Front Psychol. 2020;11: 552416. 33192801 10.3389/fpsyg.2020.552416 PMC7649279

