# Supplementary material for: The efficacy of an online exercise intervention for improving depressive symptoms among patients with subthreshold depression in primary care: protocol for a randomized controlled trial
Source: BMC Psychiatry. 2025 Apr 9;25:354. doi: 10.1186/s12888-025-06663-0 (PMC11984225; doi:10.1186/s12888-025-06663-0)
Supplement: Supplementary file 1 — Supplementary Material 1. [file 12888_2025_6663_MOESM1_ESM.docx]

**Appendix 1**

**The 12-week online EIM class structure**

| **Week** | **Content** |
| --- | --- |
| 1 | - Introduction - Breathing exercise and warm up - Low impact Chair aerobic exercise (at 50%-60% of heart rate reserve; 10 mins/ bout x 2 - bouts, with music tempo 112-120 bpm, with 3 min inter-bout rest) - Resistance exercise with body weight (major muscle group 8 reps x 2 sets) - Stretching exercise and Mind-body exercise (10 mins), Q & A |
| 2 | - Breathing exercise and warm up - Low impact Chair aerobic exercise (at 55%-65% of heart rate reserve; 10 mins/ bout x 2 - bouts, with music tempo 112-120 bpm, with 3 min inter-bout rest) - Resistance exercise with body weight (major muscle group 8 reps x 2 sets) - Stretching exercise and Mind-body exercise (10 mins), Q & A |
| 3 | - Breathing exercise and warm up - Low impact Chair aerobic exercise (at 55%-65% of heart rate reserve; 10 mins/ bout x 2 - bouts, with music tempo 112-120 bpm, with 3 min inter-bout rest) - Seated + standing resistance exercise with elastic tubes (major muscle group 10 reps x - 2 sets) - Stretching exercise and Mind-body exercise (10 mins), Q & A |
| 4 | - Warm up and stretching exercise - Low impact Chair aerobic exercise with Towel (at 60%-70% of heart rate reserve; 10 - mins/ bout, with music tempo 120-128 bpm) - Seated + standing resistance exercise with elastic tubes (major muscle group 10 reps x - 2 sets) - Stretching exercise and Mind-body exercise (10 mins), Q & A |
| 5 | - Warm up and stretching exercise - Low impact Chair aerobic exercise with Towel (at 60%-70% of heart rate reserve; 10 - mins/ bout, with music tempo 120-128 bpm) - Muscle toning exercise with dumb bell (major muscle group 10reps x 2 sets) - Stretching exercise and Mind-body exercise (10 mins), Q & A |
| 6 | - Warm up and stretching exercise - Low impact Chair aerobic exercise with Towel (at 60%-70% of heart rate reserve; 10 - mins/ bout, with music tempo 120-128 bpm) - Muscle toning exercise with dumb bell (major muscle group 10reps x 2 sets) - Stretching exercise and Mind-body exercise (10 mins), Q & A |
| 7 | - Warm up and stretching exercise - Low impact Chair aerobic exercise (at 60%-70% of heart rate reserve; 10 mins/ bout x 2 - bouts, with music tempo 120-128 bpm, inter-bout rest 3 min) - Circuit Training: dynamic muscular fitness circuit training, with small equipment (dumb - bell, elastic tubes …etc.) (20 min) - Stretching exercise and Mind-body exercise (10 mins), Q & A |
| 8 | - Warm up and stretching exercise - Low impact Chair aerobic exercise (at 60%-70% of heart rate reserve; 10 mins/ bout x 2 - bouts, with music tempo 120-128 bpm, inter-bout rest 3 min) - Circuit Training: dynamic muscular fitness circuit training, with small equipment (dumb - bell, elastic tubes …etc.) (20 min) - Stretching exercise and Mind-body exercise (10 mins), Q & A |
| 9 | - Warm up and stretching exercise - Low impact Chair aerobic exercise (at 60%-70% of heart rate reserve; 10 mins/ bout x 2 - bouts, with music tempo 120-128 bpm, inter-bout rest 3 min) - Circuit Training: dynamic muscular fitness circuit training, with small equipment (dumb - bell, elastic tubes …etc.) (20 min) - Stretching exercise and Mind-body exercise (10 mins), Q & A |
| 10 | - Warm up and stretching exercise - Chair aerobic exercise with Towel (at 65%-75% of heart rate reserve; 10 mins/ bout x 2 - bouts, with music tempo 124-132 bpm, inter-bout rest 3 min) - Circuit Training: dynamic muscular fitness circuit training, with small equipment (dumb - bell, elastic tubes …etc.) (20 min) - Stretching exercise and Mind-body exercise (10 mins), Q & A |
| 11 | - Warm up and stretching exercise - Chair aerobic exercise with Towel (at 65%-75% of heart rate reserve; 10 mins/ bout x 2 - bouts, with music tempo 124-132 bpm, inter-bout rest 3 min) - Circuit Training: dynamic muscular fitness circuit training, with small equipment (dumb - bell, elastic tubes …etc.) (20 min) - Stretching exercise and Mind-body exercise (10 mins), Q & A |
| 12 | - Warm up and stretching exercise - Chair aerobic exercise with Towel (at 65%-75% of heart rate reserve; 10 mins/ bout x 2 - bouts, with music tempo 124-132 bpm, inter-bout rest 3 min) - Circuit Training: dynamic muscular fitness circuit training, with small equipment (dumb - bell, elastic tubes …etc.) (20 min) - Stretching exercise and Mind-body exercise (10 mins), Q & A |
