# Supplementary material for: The efficacy of an online exercise intervention for improving depressive symptoms among patients with subthreshold depression in primary care: protocol for a randomized controlled trial
Source: BMC Psychiatry. 2025 Apr 9;25:354. doi: 10.1186/s12888-025-06663-0 (PMC11984225; doi:10.1186/s12888-025-06663-0)
Supplement: Supplementary file 2 — Supplementary Material 2. [file 12888_2025_6663_MOESM2_ESM.docx]

## 香港中文大學賽馬會公共衛生及基層醫療學院

**網上運動課程對改善基層醫療中的輕微抑鬱患者抑鬱症狀的效能研究：一項隨機對照試驗**

**參與者課後評估**

個案編號: 評估日期:

| 1. 這網上運動課程教授的運動對你而言有多困難？   0 1 2 3 4 5 6 7 8 9 10  非常容易 非常困難 |
| --- |
| 1. 你覺得以網上形式教授運動是否適合？   0 1 2 3 4 5 6 7 8 9 10  非常不適合 非常適合 |
| 1. 完成這網上運動課程後，你會持續運動的可能性有多大？   0 1 2 3 4 5 6 7 8 9 10  絕對不會 絕對會 |
| 1. 你會推薦這網上運動課程給患有輕微抑鬱症狀的朋友嗎？   0 1 2 3 4 5 6 7 8 9 10  絕對不會 絕對會 |
| 1. 整體而言，你覺得今次網上運動課程對你的幫助有多大？   **體能上**  0 1 2 3 4 5 6 7 8 9 10  完全無幫助 非常有幫助  如果有幫助，請列明是哪些方面：_____________________________________________________  **心理上**  0 1 2 3 4 5 6 7 8 9 10  完全無幫助 非常有幫助  如果有幫助，請列明是哪些方面：_____________________________________________________ |
| 1. 整體而言，你覺得今次網上運動課程的滿意程度如何？   0 1 2 3 4 5 6 7 8 9 10  非常不滿意 非常滿意 |
| 1. 整體而言，你認為這網上運動課程有需改善的地方嗎？   有 沒有  如有，請列明：＿＿＿＿＿＿＿＿＿＿＿＿＿＿＿＿＿＿＿＿＿＿＿＿＿＿＿＿＿＿＿＿＿＿ |
